# Supplementary material for: OsXTH19 Overexpression Improves Aluminum Tolerance via Xyloglucan Reduction in Rice Root Cell Wall
Source: Plants (Basel). 2025 Jun 22;14(13):1912. doi: 10.3390/plants14131912 (PMC12252322; doi:10.3390/plants14131912)
Supplement: Supplementary file 1 [file plants-14-01912-s001.zip › plants-3689902-supplementary.pdf]

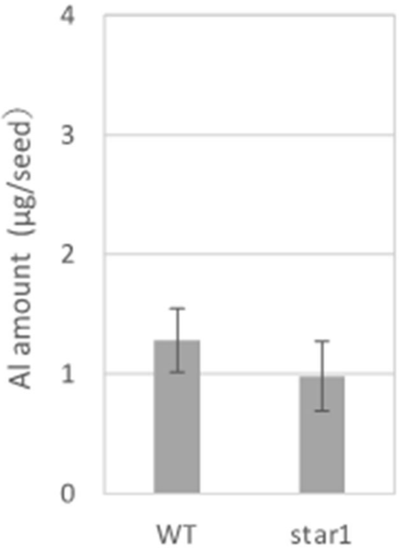

Figure S1. Al content in dry seeds of WT and the aluminum-sensitive mutant *star1*. After the extraction of Al with HNO_3, Al content was determined by inductively coupled plasma-atomic emission spectrometry. Data represent means of independent biological replicates ± standard deviation (n = 5).

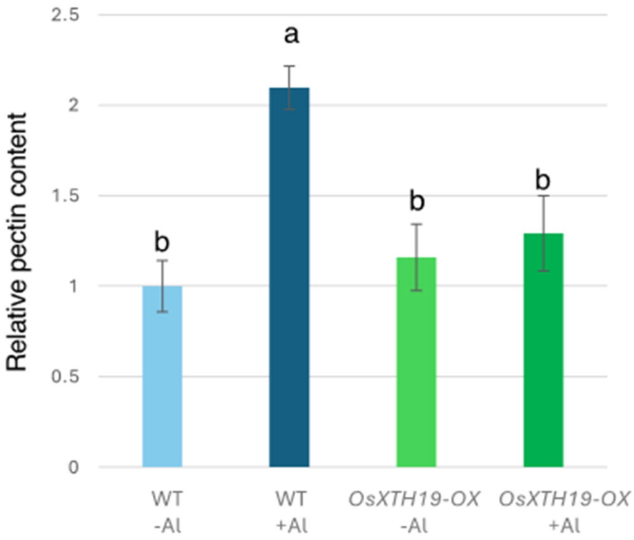

Figure S2. Relative pectin content in WT and OsXTH19-OX treated without or with Al (0 or 100 µM $AlCl_3$). The staining intensity of pectin was determined by drawing a region of interest at the area 0.1mm to 0.6mm from the root tip and measuring the mean gray value using ImageJ. The background signal, which was the same in all cases, was subtracted. Average gray values and standard deviation were determined from 5 independent biological repetitions. Different letters in each panel indicate significant differences at $p < 0.05$ (Tukey's test).
